# The Interaction among Microbiota, Epigenetic Regulation, and Air Pollutants in Disease Prevention

**DOI:** 10.3390/jpm12010014

**Published:** 2021-12-29

**Authors:** Alessandra Pulliero, Deborah Traversi, Elena Franchitti, Martina Barchitta, Alberto Izzotti, Antonella Agodi

**Affiliations:** 1 Department of Health Sciences, School of Medicine, University of Genoa, 16132 Genoa, Italy; 2 Department of Public Health and Pediatrics, School of Medicine, University of Torino, 10126 Torino, Italy; deborah.traversi@unito.it (D.T.); elena.franchitti@edu.unito.it (E.F.); 3 Department of Medical and Surgical Sciences and Advanced Technologies “GF Ingrassia”, University of Catania, 95123 Catania, Italy; mbarchitta@unict.it (M.B.); agodia@unict.it (A.A.); 4 Department of Experimental Medicine, School of Medicine, University of Genoa, 16132 Genoa, Italy; izzotti@unige.it; 5 UOC Mutagenesis and Cancer Prevention, IRCCS Ospedale Policlinico San Martino, 16132 Genoa, Italy

**Keywords:** cancer prevention, microbiota, epigenetics, environmental pollutants

## Abstract

Environmental pollutants can influence microbiota variety, with important implications for the general wellbeing of organisms. In subjects at high-risk of cancer, gut, and lung microbiota are distinct from those of low-risk subjects, and disease progression is associated with microbiota alterations. As with many inflammatory diseases, it is the combination of specific host and environmental factors in certain individuals that provokes disease outcomes. The microbiota metabolites influence activity of epigenetic enzymes. The knowledge of the mechanisms of action of environmental pollution now includes not only the alteration of the gut microbiota but also the interaction between different human microbiota niches such as the lung–gut axis. The epigenetic regulations can reprogram differentiated cells in response to environmental changes. The microbiota can play a major role in the progression and suppression of several epigenetic diseases. Accordingly, the maintenance of a balanced microbiota by monitoring the environmental stimuli provides a novel preventive approach for disease prevention. Metagenomics technologies can be utilized to establish new mitigation approaches for diseases induced by polluted environments. The purpose of this review is to examine the effects of particulate matter exposure on the progression of disease outcomes as related to the alterations of gut and lung microbial communities and consequent epigenetic modifications.

## 1. Introduction

The microbiota, present in various regions of the human body (intestine, lung, skin, etc.), is exposed to the action of environmental pollutants and contaminants present in food (preservatives, residues of antibiotics or pesticides, etc.). These environmental factors can influence microbiota variety, viability, and functionality in the long term. Some effects of the environment on the microbiota have important implications for the general wellbeing of organisms. The “microbiome”, meaning the genome of the microbiota combined with its environmental interactions, includes more than 3 million genes and is 150 times the size of the human genome [1].

Alterations of the pulmonary microbiota induced by inhalation of pollutants are related to the appearance of chronic obstructive respiratory diseases (COPDs) [2]. 

The lung microbiota can also be the target (or at least one of the targets) of the injury induced by airborne particles of assorted sizes, as well as by toxins derived from atmospheric pollution (NO, NO_2_, SO_2_, etc.) [3]. 

Exposure to elevated levels of airborne pollution increases the abundance in the lung microbiota of potential pathogens such as *Streptococcus pneumoniae* and *Neisseria* sp. [4]. It is now well known that environmental pollution represents an important risk factor for cardiovascular diseases, but the mechanisms of this effect are poorly known. Atmospheric pollution can activate local inflammatory responses as a direct effect of inhaled particles and toxic gas [5]. Local lung inflammation can become systemic due to the release of immunological and biological mediators, thus increasing the cardiovascular risk. Airborne oxidizing gaseous pollutants damage the membrane of lung macrophages in the alveoli determining the release of thromboxane from the intracellular vacuoles into the bloodstream [6]. This situation increases plasma viscosity and thrombo-philicity, facilitating platelet aggregation and clot formation, thus increasing the risk of infarction. In this scenario, similar adverse effects can be mediated by the alteration of lung microbiota, including eubiosis and dysbiosis [7]. The exact differentiation between eubiosis and dysbiosis is not yet established; however, some microbiota characteristics, such as biodiversity and ratio between microorganism groups, have been proposed [8]. Exposure to environmental pollutants, especially in early life, can lead to variations in the microbiota not only in the lung but also in the entire body, establishing a generalized dysbiosis that correlates with the incidence of a series of pathological issues at later ages such as those of an immune (such as atopic), metabolic, epigenomic, or neurological nature [9]. The maintenance of the state of wellbeing of microbiota (intestinal or otherwise) is a more complex issue than the assumption of a healthy diet rich in dietary fiber. Components of a healthy diet (such as five courses of fruit or vegetables per day) represent the preferred metabolic substrate of fermentative saprophyte intestinal bacteria allowing xenobiotic (carcinogen and pollutant) metabolization and detoxification [9,10]. Conversely, components of an unhealthy diet (such as those abundant in nitrosable substrates or amino acid pyrolysates) represent the preferred metabolic substrate of bacteria producing endogenous putrescins, mutagens, and carcinogens [11]. The knowledge of the mechanisms of action of environmental pollution should now include the alteration of the gut microbiota as well as the interaction between different human microbiota niches such as the lung–gut axis [2]. In this regard, particular emphasis should be given to new trace pollutants such as drugs, antibiotics, and disinfectants that are detected in soil, water, and air in continuously increasing amounts.

This work aims to review the existing evidence dealing with microbiota modulation and epigenetic regulation as intermediate actors between air pollution and lung cancer. The interactions between microbiota and epigenetic modulations resulting from air pollution exposure are discussed with focus on the lung–gut microbiota axis and its influence on the immune system especially during early human development. 

## 2. Early Exposure to Environmental Pollutants, and Dysbiosis as Risk Factor for Late Onset Diseases

Environmental exposure, particularly in early life, can result in the development of dysbiosis and consequent diseases [12]. Chemicals, including xenoestrogens, pesticides, and heavy metals, as well as tobacco smoking, alcohol consumption, and medical drug abuse, are major factors that unfavorably influence prenatal development and increase the susceptibility of offspring to later disease development [13]. Exposure to unhealthy lifestyle factors and environmental human-made chemical pollutants often results in the generation of reactive oxygen species (ROS) and cellular oxidative damage [14]. Oxidative stress is involved in pregnancy disorders such as abortion, intrauterine growth retardation, and prenatal mortality [15]. Upper airway microbiota assemblage begins at birth and is thus affected by the environmental exposure occurring during birth (i.e., maternal vaginal (normal birth) or skin microbiota (cesarean)). If an infant is born via cesarean, their nasopharyngeal microbiota represents their mother’s skin microbiota, whereas if born via the vaginal route, their microbiota will resemble the maternal urogenital microbiota [16]. Positive and negative changes to the microbiota may be attributed to intramicrobial interaction and the immune response to a pathogen [17]. Influenza A infections modify the lung microbiota by increasing the presence of pathogenic bacteria. Probiotics are a promising therapeutic for dysbiosis and are used in many diseases, such as asthma. Distinct nasopharyngeal microbiota predicts the risk and severity of asthma-related inflammation [18]. During the first year of life, increased nasopharyngeal colonization of *Streptococcus sp*. occurs. Individuals suffering from obstructive sleep apnea have a distinct nasal microbiota, the microbial diversity and composition distinctions in patients correlating with inflammatory biomarkers. A recent study in children presenting SARS-CoV-2 infection demonstrated that both the upper respiratory tract and the gut microbiota were altered. The alteration of the microbiota in these children was dominated by the genus *Pseudomonas* and remained altered up to 25–58 days in different individuals [19]. As children do not experience the same complications associated with COVID-19 as adults do, these microbiota profiles give important insight into the role of the microbiota in disease susceptibility. The pollution and prolonged stress change the balance of the systems, in particular the immune system, which obviously dialogues with all other systems. In fact, the basic condition common to many diseases, including cancer, is mild and silent but chronic systemic inflammation.

Insults provided by pollutants—from motor vehicles, incinerators, particulate matter, heavy metals, pesticides, or electromagnetic fields—are more harmful if the exposure occurs during gestation or in the very first years of life. At this point the epigenetic programming takes place that even 10 or 20 years later or in subsequent generations could lead to serious pathologies [20]. The entire genome is a unitary, dynamic, fluid, and systemic molecular network in a continuous relationship with the environment. The flow of information from the outside meets the information that has been encoded for millions of years in DNA, organizing the main molecular processes that determine the structure and functions of cells and tissues. Thus, the human microbiota is constantly changing because of the information it receives from the outside resulting in a physiological (or pathological) adaptive reactivity. 

### The Microbiota and Epigenetic Regulation

Since the beginning of their evolution, humans have lived in constant association with bacteria. The number of bacteria in the human body exceeds the number of human cells. The bacteria genome (metagenome) is about 100 times the size of the human genome. The total weight of the bacteria contained in our body is about 1.5 kg. This massive bacteria presence has been neglected by research for many years and its importance underestimated in the therapeutic protocols [21].

The microbiota is present in five macro-areas of the human body, all in continuity with the outer environment, oral nasal cavity, skin, and gastrointestinal and urogenital tracts. Ongoing research programs aim at sequencing the metagenome, examining the relationship between bacterial species and human health by computational analyses. About 70% of the bacteria composing the human microbiota is in the gastrointestinal tract, with a concentration increasing in an exponential way in the oral–fecal sense. Bacteria colonization happens at the moment of birth, and the initial pattern of bacteria depends on the type of birth [22], even if such diversity disappeared early. Indeed, a natural birth allows high number of maternal bacteria to be transmitted to the newborn, while this situation does not occur in the cesarean delivery [23]. 

From the first 4 weeks of life onwards, especially after weaning, the composition of the bacterial microbiota tends to be pretty stable but may remarkably vary in case of pathological conditions. The intestinal bacteria perform important metabolic functions: (a) digestion of non-digestible carbohydrates, (b) production of short-chain fatty acids representing an energy source for bacteria and intestinal epithelial cells and regulating the sensitivity to insulin, (c) acidification of the intestinal lumen limiting the formation of endogenous mutagens such as putrescins and nitrosable amines, (d) physiological maintenance of the intestinal motility, (e) production of vitamins of group B (pantothenic acid, pyridoxine, and riboflavin) and biotin, (f) participation in the transformation and re-adsorption of bile, and (g) synthesis of amino acids. The microbiota can be seen as a tuned metabolic “organ” of our physiology [24]. It also performs protective functions and increases the barrier effect increasing the production of mucin and zoludine, a component of the tight junctions allowing the intestinal epithelium to perform a protective barrier function. 

Nutrition is an acceptable intervention opportunity which plays a key role in many aspects of health. Food imbalances are the main determinants of chronic diseases, including cardiovascular disease, obesity, diabetes, and cancer. Many epidemiological and experimental data show that suboptimal early nutrition can have consequences for health even several decades later, supporting the hypothesis that epigenetic mechanisms form the link between nutritional imbalances and disease risk [25]. Of course, diet is one of the factors that most affects the variability of genetic expression since, in addition to a direct biochemical action of nutrients, it determines the composition of the microbiota, especially the intestinal one. However, it is only one of the complex modulators of health that has been studied under an exposome approach, including external and internal factors. Accordingly, the microbiota represents a dialogue interface between the environment and the host.

The microbiota affects host health by regulating epigenetic mechanisms such as host microRNAs (small non-coding RNAs), chromatin dynamics, and histone modifications [26] as well as DNA methylation [27].

In pregnant women an association was revealed between bacterial predominance and epigenetic profile. In particular, in mothers in which *Firmicutes* bacteria were dominant, methylation profile analysis carried out in blood samples found 568 hypermethylated genes and 254 hypomethylated genes, some of which are associated with the risk of cardiovascular disease, lipid metabolism, obesity, and inflammatory response [28]. In mice, microbial intestinal colonization of the mother may alter epigenetic signatures of the gut establishing an inflammatory environment predisposing to the delivery of premature infants. The same study analyzed intestinal bacteria in mice at 2 weeks of life showing that 16S RNA sequencing conditioned early microbiota colonization leading to differential bacterial colonization at different taxonomic levels [29].

The microbiota amplifies our adaptive capacity as it changes quickly in relation to the environment and protects us from environmental changes. Microbiota colonization by pathogenic bacteria such as *Helicobacter pylori* and *Klebsiella* sp. influences the methylation patterns of the host. Individuals with *Helicobacter pylori* infection display very high methylation levels in several CpG islands in the gastric mucosa, this finding indicating that the infection alters DNA methylation [30]. Different studies have shown that changes in the gut bacterial composition can alter methylation and inhibit histone deacetylases [31]. 

The microbiota metabolites influence activity of epigenetic enzymes. For example, butyrate, a metabolite produced in case of dysbiosis, inhibits histone deacetylases increasing the expression of the FOXP3 gene through the acetylation of histone H3 in its promoter [27,32]. Environmental exposure during the first years of life can induce persistent alterations in the epigenome thus leading to an increased risk of obesity later in life. This also means that it is feasible to predict the risk of obesity of an individual at a young age by analyzing their intestinal microbiota. This prediction is the prerequisite for targeted prevention strategies modifying unfavorable epigenomic and microbiota profiles, starting from the lifestyle of the pregnant woman and then continuing during adulthood and ageing [33]. 

## 3. Air Pollution Can Modify the Intestinal Microbiota

The development of techniques based on the sequencing of the 16S subunit of ribosomal RNA, allowing the detection of “living” and “non-living” bacteria, has facilitated the identification of the metagenome, that is the complex superorganism consisting of the microbiota and the host genome [34]. This ecosystem, in which billions of bacteria coexist and interact with the host organism, is capable of (a) regulating many systemic functions; (b) contributing to the state of health; (c) playing a role in gastrointestinal diseases (irritable colon, chronic inflammatory colitis, diverticulitis, colon cancer); and (d) playing a role in systemic diseases (allergies, obesity, metabolic syndrome, type 2 diabetes, atherosclerosis) [35].

Air pollution seems to be able to modify the composition and the function of the human gut microbiome even if the mechanism of action is not yet clearly understood. A recent study reported that inhalation for 24 h of high levels of ozone gas was associated with *Bacteroides caecimuris increase* in gut microbiota and alteration of multiple gene pathways in the microbiome. Conversely, exposure to high nitrogen oxide was associated with *Firmicutes* increase in gut microbiota [36]. 

**Figure 1:**
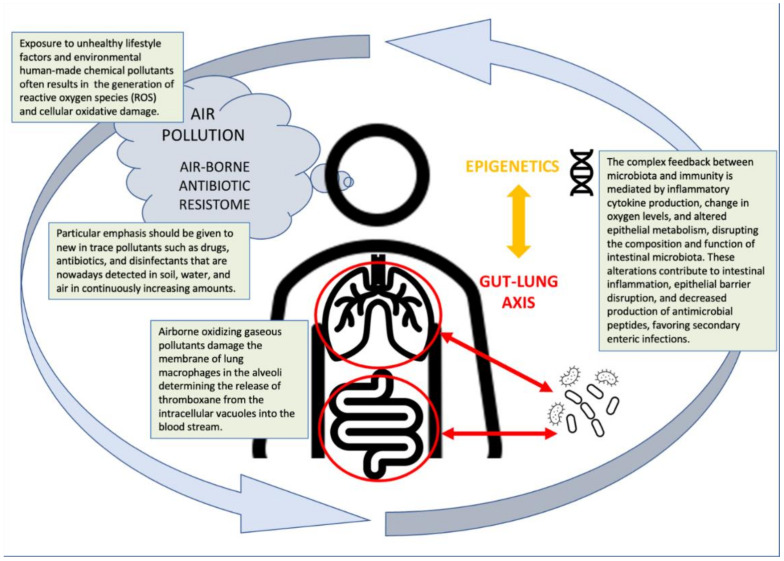
How the environment affects the lung–gut axis of microbiota.

The percent of variation in gut bacterial composition that was explained by exposure to air pollution was up to 11.2% for ozone, thus identifying this pollutant as able to alter the human gut microbiota [37]. A significant association between exposure to air pollutants and gut microbiome alterations was reported in young adults residing in Southern California, identifying inhalation of ozone gas as an important pollutant that may alter the human gut microbiome [37]. They found that 128 bacterial species were associated with inhalation of ozone gas, and four and five bacterial species were associated with inhalation of nitrogen oxides. Various atmospheric pollutants have been associated with modifications of gut microbiota in humans. A positive correlation was shown between the abundance of the *Micrococcus* and *Actinobacteria* and exposure to high levels of polycyclic aromatic hydrocarbons (PAHs) such as dibenzo (a, h) anthracene and indeno (1,2,3-cd) pyrene. Accordingly, PAH exposure may disturb metabolic pathways (such as metabolism of purine, pyrimidine, lipid, and folate) through imbalance of commensal microbiota. Two studies have shown that exposure to nitrogen oxide near roadways correlated to an increase in gut bacteria associated with obesity and altered metabolism [38]. A recent population-based epidemiological study found that the gut microbiota partially mediates the effect of fine particulate matter (PM) on the risk of developing type 2 diabetes [7]. Studies suggested that air pollutants can adversely affect the gastrointestinal tract [39], where ultrafine particles can reach the intestine through inhalation and diffusion from the terminal alveoli into the systemic circulation or through the ingestion of inhaled particles after mucociliary clearance from the airways to the oropharynx [39,40,41]. Once in the gut, PM components can alter the composition and the function of the gut microbiota selecting the growth of specific bacteria [42,43,44]. PM 2.5 and inhaled ozone gas have been shown to have extrapulmonary effects that can alter the hypothalamic–pituitary–adrenal axis through vagal nerve activation [45] or effects on the hippocampus [46] thus increasing the levels of catecholamines and steroid hormones. Recent studies revealed a link between PM and gastrointestinal diseases including appendicitis [47] and colorectal cancer [48]. In PM-exposed mice, increases of gut microbiota diversity in the small bowel, colon, and feces and alterations of the gut microbiota composition along the gastrointestinal tract have been reported [49]. Experimental studies have indicated that alterations in the gut microbiota play a role in the pathway of diabetes induction resulting from particulate matter pollution with aerodynamic diameters <2.5 μm (PM 2.5 was positively associated with the risks of impaired fasting glucose (IFG) and type 2 diabetes and negatively associated with alpha diversity indices of the gut microbiota [50] (Figure 1, Table 1).

## 4. The Lung–Gut Axis and the Influence of the Microbiota on the Immune System

Intestinal microbiota modifications can modulate disease outcomes in the gut but also in distant organs as demonstrated in animal models by experimentally transferring of dysbiotic microbiota [53,54,59,60].

The communication between the gut and other organs and tissues is mainly mediated by microbial metabolites and immunity modulation [61] 

Gut and lung microbiota are different, both in terms of abundance and in terms of composition, even if there are some structural and functional similarities between lung and gut epithelium [62]. The different composition is due to the existing differences in oxygen availability. One of the most relevant similarities is the ability to interact with the immune system in conjunction with associated lymphoid tissue [63].

The lungs have a large surface area with high environmental exposure. In healthy lungs, microbial DNA was detected. Microorganisms probably reached the lungs from the oral cavity through microaspiration, as the taxonomic profiles of the two sites were quite similar. Comparing the two profiles, the lung microbiota had a decreased abundance of *Prevotella* spp. and an enrichment of Enterobacteriaceae, *Ralstonia* spp., and *Haemophilus* spp. with respect to the oral cavity microbiota [64]. 

The lung microbiota differential genera in healthy individuals with respect to COPD and lung cancer patients are mainly *Moraxella*, *Haemophilus*, *Streptococcus*, *Pseudomonas*, *Staphylococci*, *Veillonella*, *Enterobacter*, *Escherichia*, and *Megasphaera* [65] 

A lower lung microbiota alpha diversity was observed in subjects with a higher exposition to air pollutants [38]. Moreover, variance in both the respiratory microbiota [66] and gut microbiota [67] was observed also in relation to air pollution exposure.

The gut microbiota metabolites can reach distant organs including both the lungs and particularly protected organs such as the brain [68]. Promising research frontiers include psychobiotics development as a complementary treatment for depression or other mental illness and personalized care protocols considering the genetic and microbiome patient characteristics for example in chemotherapy treatment [69]. Alterations in the microbiota can also modulate host behaviors such as social activity, stress, and anxiety-related responses that are linked to diverse neuropsychiatric disorders [70]. Indeed, some researchers demonstrated that manipulation of the microbiota in either antibiotic-treated or germ-free adult mice results in significant deficits in fear extinction learning [71,72]. After birth, the microbiota strictly influences the host’s immune system maturation. A range of hypotheses exist for disease pathogenic pathways [64] such as for T1DM [71,72,73] and (Bowel Inflammatory Disease) IBD [74]. All such models include an altered stimulation of the host immune system by the microbiota [75]. 

The complex feedback between microbiota and immunity is mediated by inflammatory cytokine production, change in oxygen levels, and altered epithelial metabolism, disrupting the composition and function of intestinal microbiota. These alterations contribute to intestinal inflammation, epithelial barrier disruption, and decreased production of antimicrobial peptides, favoring secondary enteric infections. 

Recent evidence on the respiratory diseases showed that gut dysbiosis due to viral respiratory infection also results in diminished production of microbial associated protective molecular patterns including toll-like receptor and microbial metabolites such as SCFAs, thus reducing antibacterial pulmonary immunity. Such lung–gut interconnections might be particularly relevant during SARS-CoV-2 infection [76], especially when associated with other weakening of the lung defenses due to high level of air pollutants [77]. However, the experimental data are fast increasing on lung–gut interaction, and one of the main questions is how to clear a causation or an association relationship, also considering the role of the air pollution exposure of the host. 

CO_2_, SO_2_, and other toxic gases and airborne particulate matter (PM) constitute a universal danger to exposed organisms. Correlations of long-term exposure to air pollution and mortality have been addressed in different studies worldwide [78]. The existence of an association between long term exposure to fine PM and an increased risk of cardiovascular and lung disease, as well as lung cancer, has been established [79] Air pollution is also associated with gastrointestinal disorders and inflammatory bowel diseases [80]. Where inhaled particles are deposited in the respiratory system depends on their size. Most of the larger particles are sequestered in the upper airways such as trachea and large bronchi [81]. Smaller size particles, particularly PM 2.5 or less, can reach the bronchioles and alveolar spaces where they are phagocytosed by alveolar macrophages [49]. Particles sequestered in macrophages and directly in the mucus layer in lower airways are subsequently transported up to the oropharynx and then swallowed into the gastrointestinal tract [82]. PM can also be ingested directly by consumption of food and water contaminated by PM [36,41,74,75,83]. It has been estimated that 10^12^–10^14^ particles are ingested per day by an individual on a Western diet [84]. Treatment of gut epithelial cells with PM caused increased production of mitochondrial reactive oxygen species (ROS), release of inflammatory cytokines, and induced apoptosis of colonocytes [37]. Several studies suggested that smoking suppresses the innate immune response to bacteria through the direct inhibition of bacterial sensing patterns such as the recognition of lipopolysaccharides by the TLR4/MD-2 receptor. Smokers with active Crohn’s disease were reported to have a clinically relevant dysbiosis of the gut microbiota [85]. In mice, high-fat and fiber-deprived diets change the composition of intestinal microbiota and damage the intestinal barrier through increased intestinal permeability, reduced thickness of the mucous layer, abnormalities of tight junction proteins of the epithelial barrier, and low-grade intestinal inflammation [41]. A variety of environmental factors, such as diet and PM exposure can influence H_2_S regulation and function. Epigenetics also have a role in H_2_S regulation. In addition, new research into the role of gut microbiota in the development of hypertension has highlighted the need to further explore these microorganisms and how they influence the levels of H_2_S throughout the body affecting the microbiota [41].

In bronchoalveolar lavage cells, tobacco smoke exposure increased the activity of inflammatory pathways by inducing continuous active demethylation processes [86]. 

Exposure of human macrophages to cigarette smoke extract also promoted pro-inflammatory cytokine release by activation of the NF-κB pathway and concomitant posttranslational modifications of HDACs [87].

## 5. Metagenomics Approaches to Study Microbial Communities 

Metagenomics is a set of research techniques, comprising many related approaches and methods, to understand the genetic composition and activities of microbial communities so complex that they can only be sampled, never completely characterized. The use of new high-throughput technologies is driving microbiology from an approach predominantly focused on the study of single species in pure laboratory culture into a new era focused on the characterization of whole microbial communities. Metagenomics involves the characterization of the genomes in these microbial communities, as well as their corresponding messenger RNA, protein, and metabolic products. Thus, metagenomics combines the power of genomics, bioinformatics, and systems biology to analyze the genomes of many organisms simultaneously. Particularly, in metagenomics studies, DNA is extracted directly from all the microbes living in a particular environment, and the mixed DNA sample is analyzed, using different high-throughput DNA sequencing approaches and computational methods, in order to create a plethora of metagenomic library/datasets that contain the genomes of all the microbes found in that environment [88]. This can be used to analyze the microbial diversity, population structure, evolutionary relationship, functional activity, and the relationship between community and environment [89] and is one of the best technological innovations to improve bioremediation strategies. Metagenomic datasets from different microbial ecosystems can also be compared to uncover the traits that are important to each ecosystem [90]. Metagenomics can address several potential prospects in different areas ranging from life and biomedical sciences to environmental biotechnologies, agriculture, and microbial forensics. Furthermore, metagenomics has most frequently been utilized to study the microbial communities capable of degrading hydrocarbon and thus establishing new mitigation approaches for polluted environment [91]. 

In 2007, the National Institutes of Health (NIH) launched the Human Microbiome Project (HMP) in order to study and characterize the microbiome and the factors that influence the distribution and evolution of the microbiota. The project aims to identify new diagnostic biomarkers for health applications and a deeper understanding of the nutritional requirements of humans to drive new recommendations for food production, distribution, and consumption [92]. 

Over the past decade, numerous technologies have been developed for analyzing microbial community structure and functions. In traditional techniques, cultivation-based methodologies and phenotypic characterization were used to describe the diversity of microorganisms in the studied samples. Although amplicon sequencing, as the PCR-based 16S rRNA analysis, is the most widely used method for characterizing the diversity of microbiota, these methods, also referred to as metataxonomic, have some limitations. For example, novel or highly diverged microbes are difficult to study using this approach since sequencing is limited to the analysis of taxa for which taxonomically informative genetic markers are known. In any case metataxonomic methods, requiring sequences from a single gene, provide a cost-effective means to identify a wide range of organisms. Limitations of this approach have been addressed by the development of metagenomic analysis that uses sequencing, and now high-throughput sequencing and microarray technologies—“open-format” and “closed-format” detection technologies, respectively—combined with high-performance computational tools, to provide information on the species composition of a microbiome [15]. In recent years, next generation sequencing technology has been used to rapidly and efficiently profile whole microbial communities in various samples, revolutionizing genome research because of its capability to produce a large quantity of sequence data in a relatively short period of time [93]. Shotgun metagenomic sequencing, a relatively new and powerful sequencing approach that uses the random sequencing of all genomic content of a microbiome, allows researchers to measure all genes in all organisms present in the community of the study sample, overcoming many of the limitations of amplicon sequencing. Shotgun metagenomics also provides a means to study unculturable microorganisms and to study biological functions encoded in the genomes of the organisms that make up the community [94]. In a recent study, results obtained by the metataxonomic approach and metagenomics were compared to investigate their reliability for bacteria profiling. The results showed that 16S rRNA gene sequencing detects only part of the gut microbiota community revealed by shotgun sequencing, and interestingly, the less abundant genera detected only by shotgun sequencing are biologically meaningful [95]. However, targeted and shotgun sequencing of DNA cannot distinguish between expressed and nonexpresser genes in a given environment. Thus, new meta-transcriptomic sequencing approaches have provided insight into microbial community functions and activities from diverse habitats in understanding how a microbial community responds over time to its changing environmental conditions [96]. 

## 6. Environmental Antibiotic Pollution and Microbiota: Implication for Public Health 

**Figure 2:**
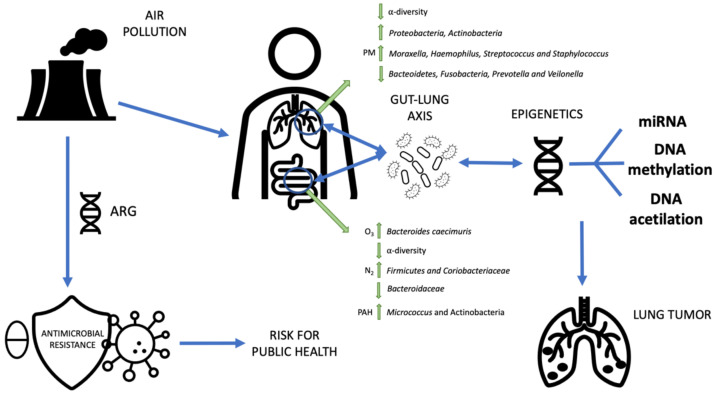
Bidirectional communication between the lung and gut microbiota. The impact of environmental pollution, including the airborne resistome, on human lung health is influenced by the lung–gut axis microbiome and epigenetic regulation. The green arrows represent the microorganisms that change after exposure to air pollution [31,32,33,34,35,36,37,38,39,40,41,42,43,44,45,46,47,48,49,50,51,52,53,54,55,56,57].

Antimicrobial resistance (AMR) is one of the top 10 global public health threats facing humanity that requires urgent multisectoral action to achieve the Sustainable Development Goals (SDGs). AMR occurs naturally, and antimicrobial resistant organisms and antimicrobial resistant genes (ARG) are found in humans, animals, food, plants, and in the environment, including water, soil, and air [15]. However, one of the most reported consequences of the widespread overuse and inappropriate usage of antibiotics is the increased frequency of bacteria harboring ARGs in different environments, now referred to as “antibiotic resistance pollution” [97]. Since ARGs may cause consequences for human health, understanding their occurrence would be of great public health interest and value. New technologies such as next generation sequencing and metagenomics approaches allow the real-time monitoring of antimicrobial resistant organisms and ARGs in the environment and have the potential to detect microbial reservoirs and transmission routes [98] in order to prevent the increase and the spread of AMR with consequences for human health. Recently, due to an emerging public health concern, airborne ARGs carried by antimicrobial resistant organisms found in urban air have received more attention. Interestingly, it has been reported that the quantity of ARGs inhaled via airborne fine particulate matter (PM 2.5) was equivalent to that ingested via water intake [99]. A recent article reports results of a global metagenomic map of urban microbiomes and antimicrobial resistance in 60 cities across the world identifying 4246 known species of urban microorganisms, a set of 31 species distinct from human commensal organisms and an irregular distribution of AMR genes across cities that could be the result of different levels of antibiotic usage or reflect the background microbiome in different places in the world [100]. A global survey of ARG abundance in air conducted across 19 world cities demonstrated that urban air had been polluted by several ARGs and that different cities are challenged with health risks due to airborne ARG exposure [101]. The study of Hu et al., using publicly available metagenome sequences characterized the diversity and abundance of ARGs in the PM during a severe smog event in Beijing and revealed that both the abundance and diversity of airborne ARGs were higher in smog days than in non-smog days [102]. In another recent study using a high-throughput sequencing approach, profiles of ARGs were obtained from PM2.5 and PM10 sampled in four seasons for one year from a general hospital, the urban community near the hospital, and the nearest suburban community in China. In total, 643 ARG subtypes belonging to 22 different ARG types were identified. The hospital exhibited higher ARG abundance and was more closely associated with clinically important pathogens than the nearby communities [103]. In conclusion, the availability of environmental microbiome and ARG characterization and of metagenomic maps could provide the opportunity to generate significant evidence on the impact of environmental antibiotic pollution and microbiota on human health and give tools to public health authorities to assess risk, map outbreaks, and predict epidemiological risks and trends [104]. Understanding and fighting antibiotic resistance pollution using a “One Health Approach”, in which multiple sectors—public health, animal health, plant health, and the environment—work together to achieve better public health outcomes, may aid in creating more societal engagement and ultimately more efficient policies to evaluate direct risks of transmission posed by certain contaminated environments [105] (Figure 2). 

## 7. Conclusions

The microbiota is considered a “system” that carries out various vital functions in our bodies. Many factors are involved in the normal functioning of this organ system of the body which leads to microbial dysbiosis. This not only alters the composition of microbial communities but also leads to alteration in normal physiological functioning associated with normal microflora. Alteration in the composition and function of the gut microbiota has a direct effect on human health and plays an important role in the occurrence of several diseases. 

In this review, we discuss various host and environmental factors that significantly influence the biodiversity of microbiota. There is evidence suggesting that respiratory infections not only alter the lung microbiome but also promote signals of infection from the lungs to the gut with consequent alterations in the gut microbiome [106]. During a respiratory infection, bacteria and immune cells can translocate across lung epithelial cells and reach the gastrointestinal tract via lymphatic or blood circulation to activate local intestinal immunity. The microbiome of the lung and the gut have been implicated in environmentally determined diseases. The symbiosis between the microbiota and its mammalian host encompasses multiple relationships [107]. The capacity of a given microbe, including those composing the microbiota, to trigger or promote disease is highly contextual, and some microbes can shift from mutualist to commensal to parasite according to the state of activation of the host, coinfection, or localization [108]. These results demonstrate the level of communication between the gut and the lungs in response to alterations in the intestinal microbiota and intestinal permeability. The lung–gut axis is a two-way system that involves interactions between the respective microbiota and immune cells. There has been growing evidence of host–microbe and microbe–microbe interactions that shape immune responses in respiratory diseases and the development of subsequent effects in the gut. Environmental insults induce these imbalances but environ-mental exposure has also been identified to protect against allergies, foster in particular microbiome diversity, and contribute significantly to barrier organ functioning. Pollutants induce oxidative stress and inflammation, genomic and epigenetic alterations, mitochondrial dysfunction, altered intercellular communication, and altered microbiome communities. Taken together, they provide a framework for understanding how environmental insults, even at relatively low concentrations, can manifest chronic diseases. Advances in biomedical technologies will elucidate the complex interplay of environmental insults down to the single cell level. There is an important potential for harnessing the understanding of the links between environmental insults and health to propose individualized prevention and treatment strategies. As reviewed herein, experimental studies to date provide evidence that exposure to environmental pollutants triggers alteration of the human microbiome. Still pending is the possibility of preventing, or at least attenuating, these alterations by preventive approaches such as diet modification, dietary integration, oral administration of probiotics, and fecal transplant. Further studies are required to evaluate whether or not these approaches may represent a new strategy in protecting the human organism form environmental pollutants. 

The variability of experimental conditions combined with the presence of mixtures of emerging contaminants as well as the epigenetic effects constitute the main challenges to be overcome for prioritization of “One Health” environmental pollutants. 

## Figures and Tables

**Table 1:** An overview of studies focused on associations between particulate matter, gut, and lung microbiota alteration.

Particulate Matter	Microbiota	References
PM2.5 exposure in mice	Lung/intestinal damage and systemic inflammatory reactions	[51]
Inhaled diesel PM2.5 in mice	Alteration of gut microbiota diversity and community	[7]
PM can be indirectly deposited in oropharynx via mucociliary clearance and upon swallowing of saliva and mucus	Alteration of the GI epithelium and gut microbiome	[49]
Antibiotics, air pollutants, lifestyle, diet, breast feeding	Mucosal inflammation	[52]
Particulate matter, nitrogen oxides, and ozone	Alteration of the gut microbiota with risk of obesity and type 2 diabetes	[53]
Traffic-related air pollution	Gut microbial taxa and fasting glucose levels	[38]
Polycyclic aromatic hydrocarbons (PAHs)	Modulation of endocrine signaling pathways in gut microbiota	[54]
Particulate matter (PM)	PM-induced neutrophilia	[55]
Air pollution	Increased risk of metabolic dysfunction in obese individuals	[56]
Particulate matter including diesel exhaust particles	At relevant doses, changes the composition and function of the gut microbiota	[57]
Particulate matter	Promote Pseudomonas aeruginosa infection	[50]
Particulate matter	multiple gastrointestinal symptoms in patients with COVID-19 and progression with special emphasis on the lung–gut axis	[58]
